# Sensitive fluorescent hybridisation protocol development for simultaneous detection of microRNA and cellular marker proteins (in the retina)

**DOI:** 10.1007/s00418-018-1705-6

**Published:** 2018-08-07

**Authors:** Andrea Kovács-Valasek, Bálint Szalontai, György Sétáló, Robert Gábriel

**Affiliations:** 1 Department of Experimental Zoology and Neurobiology, University of Pécs, Ifjúság útja 6, Pécs, 7624 Hungary; 2 Department of Medical Biology, University of Pécs, Pécs, Hungary; 3 János Szentágothai Research Centre, University of Pécs, Pécs, Hungary

**Keywords:** MicroRNA, In situ hybridisation, Immunocytochemistry, Tyramide signal amplification, mir-9, mir-23

## Abstract

Nowadays, increasing number of microRNAs are found to have crucial roles in various physiological processes through gene expression regulation via RNA silencing as a result of base pairing with complementary mRNA sequences. To reveal the spatial distribution of microRNA expression in tissues, in situ hybridisation is the only method developed to date. This work aims to provide a novel approach to obtain information on the possible involvement of microRNA-s in regulatory processes under experimental conditions by enhancing fluorescent detection of microRNA labelling. Developing Wistar rats were used as a model system to analyse retinal microRNA expression in the first 3 postnatal weeks. Using cryosections, the crucial elements of optimal labels were (1) the concentration and duration of proteinase K treatment, (2) hybridisation temperature of microRNA probes and (3) temperature of stringency washes. Further improvements made possible to combine our in situ hybridisation protocol with double-label immunofluorescence allowing for the simultaneous detection of microRNA-s with high sensitivity and a neuronal cell marker and/or a synaptic marker protein. Thus, the regulatory microRNA-s can be localised in an identified cell type along with its potential target protein. We believe that our protocol can be easily adapted for a variety of tissues of different origins, developmental stages and experimental conditions.

## Introduction

MicroRNA-s generate a well-controlled, fine-tuning regulatory network which could play crucial roles in various physiological processes through RNA silencing by base pairing with complementary mRNA sequences (Bartel 2004). They have generated considerable interest in the field of neurobiology especially in neural development, aging and neurodegeneration (Kalani et al. 2014; Barmada 2015). These small molecules are only 20–25 nucleotides long, and are able to bind to multiple targets (Barter et al. 2015). Although individual microRNA-s may have multiple targets, in several cases they play a well-defined role in the case of an individual cell type and target protein regulating expression level and sometimes even cell fate (Bartel 2004). Organ-specific microRNA maps have also been prepared (Karali et al. 2010; Diaz et al. 2015; Zhou et al. 2016) to monitor developmental or pathological processes at this regulatory level.

There are several opportunities to examine the expression of the microRNA-s. While qPCR, microRNA array or RNA-Seq analysis are common techniques for monitoring the quantity and even more the qualitative profile of microRNA-s in an appropriate timeframe, in situ hybridisation (ISH) is the only method that could provide evidence about localization of microRNAs within organisms, organs, tissues or cells (Obernosterer et al. 2007; Mohr and Mott 2015). However, the spatial resolution offered by conventional microRNA hybridisation is rather poor (Ryan et al. 2006; Karali et al. 2010). Since the emergence of *T*_m_-normalized, ultra-sensitive locked nucleic acid (LNA) probes much more information have become available on spatial distribution of microRNAs (Obernosterer et al. 2007; Thompson et al. 2007; Kasai et al. 2016). However, these seminal works have mainly focused on the detection of highly expressed brain microRNAs. At the same time revealing low-abundance of microRNA-s or microRNA-s from other than brain tissues could still prove challenging for researchers and always requires time-consuming optimisation steps in each laboratory. In our laboratory, we perform experiments on retinal development, aging and metabolic retinal degenerations (e.g. Szabadfi et al. 2014; Kovács-Valasek et al. 2017; Lakk et al. 2017). All these aspects may potentially involve microRNA regulation (Mortuza et al. 2014; Rasheed et al. 2014; Smit-McBride et al. 2014; Chung et al. 2015). Therefore, this paper aims to improve our ability to obtain widespread information on microRNA regulation under experimental conditions by enhancing fluorescent detection of microRNA-s. We also describe how in situ hybridisation can be combined with (single or double labelling) immunocytochemistry.

## Materials and supplies

### Animals

In the present study developing Wistar rats were used as a model system to analyse retinal microRNAs in the first 3 postnatal weeks (P7, *n* = 3; P10, *n* = 5; P15, *n* = 5; P21, *n* = 3). All the animal care and handling procedures were performed according to the ethical guidelines, approved by University of Pécs Animal Ethics committee (BAI/35/51–58/2016). The rats were kept on 12 h light/dark cycle. Food and water were available ad libitum.

### Equipment and supplies

The following equipment and supplies were used in the course of our study: CM1860 UV Cryostat (Leica, Wetzlar, Germany); Super Frost Ultra Plus glass slides (Thermo Fisher Scientific, Budapest, Hungary); SI-1202 Enviro-Genie Scientific Industries Inc. incubator (Scientific Industries, Bohemia, USA); glass beakers; Olympus IX 81 inverse platform—Olympus Fluoview FV-1000 Laser Confocal Scanning Microscope (Olympus, Tokyo, Japan); Adobe Photoshop 7.0 program for image processing.

### Buffers and solutions

For RNA protection diethylpyrocarbonate (DEPC; Sigma-Aldrich, Budapest, Hungary) and RNase Away (Thermo Fisher Scientific) solutions were used. For preparing fixatives and buffer solutions sodium hydroxide (Sigma-Aldrich), paraformaldehyde (PFA; Merck, Budapest, Hungary), sucrose (Sigma-Aldrich), triethanolamine (TEA; Sigma-Aldrich), acetic anhydride (Sigma-Aldrich), sodium chloride (Sigma-Aldrich), ethylenediaminetetraacetic acid (EDTA; Sigma-Aldrich), disodium hydrogen phosphate dehydrate (Na_2_HPO_4_ × 2 H_2_O; VWR), sodium dihydrogen phosphate anhydrous (NaH_2_PO_4_; Spektrum 3D, Pécs, Hungary) and trisodium citrate dihydrate (Reanal, Budapest, Hungary) were utilised. Denhardt’s solution (Thermo Fisher Scientific) and concentrated hydrochloric acid (HCl; Reanal) were also purchased for this purpose. Shandon^™^ Cryomatrix (Thermo Fisher Scientific) was the embedding material for sectioning while proteinase K (Thermo Fisher Scientific) pretreatment provided proper reagent penetration. For hybridisation formamide (Sigma-Aldrich), yeast RNA (Roche, Budapest, Hungary), and 5′-DIG-labeled LNA probe were used. Signal detection utilised anti-digoxigenin (mouse)-horseradish peroxidase (anti-DIG HRP) conjugate (Perkin Elmer, Per-Form Hungary Kft., Budapest, Hungary) and tyramide signal amplification (TSA) fluorescence kit (Perkin Elmer). All information on chemicals are collected in Table 1, and all the immune and hybridisation reagents in Table 2.

**Table 1:** Chemicals used during the protocols

Chemicals	Company	Order numbers	Stock solution	Working solution
Acetic-anhydride	Sigma-Aldrich, Budapest, Hungary	320102	99%	0.2%
Diethylpyrocarbonate (DEPC)	Sigma-Aldrich, Budapest, Hungary	D5758	97%	0.1%
Formamide	Sigma-Aldrich, Budapest, Hungary	F9037	99.5%	50%
Hydrochloric acid (HCl)	Reanal, Budapest, Hungary	30715-0-0169	37%	Used for pH adjustment
Triethanolamine (TEA)	Sigma-Aldrich, Budapest, Hungary	90279	99%	10%
Ethylenediaminetetraacetic acid (EDTA)	Sigma-Aldrich, Budapest, Hungary	E5134	Pure	0.5M
Di-sodium hydrogen phosphate dehydrate (Na_2_HPO_4_ × 2 H_2_O)	VWR, Hungary	10028-24-7	Pure	7.1%
Paraformaldehyde (PFA)	Merck, Budapest, Hungary	30525-89-4	Pure	4%
Sodium-dihydrogen phosphate anhydrous (NaH_2_PO_4_)	Spektrum 3D, Pécs, Hungary	06090543	Pure	2.4%
Blocking reagent	Roche, Budapest, Hungary	11096176001	Pure	0.5%
Sodium-chloride	Sigma-Aldrich, Budapest, Hungary	71380	Pure	Used in different concentration for different solutions
Sucrose	Sigma-Aldrich, Budapest, Hungary	84097	Pure	15% and 30%
Trisodium-citrate dihydrate	Reanal, Budapest, Hungary	34761-1-08-38	Pure	0.3 M
Yeast RNA	Roche, Budapest, Hungary	10109495001	Pure	0.5 mg/ml
Sodium-hydroxide sol	Sigma-Aldrich, Budapest, Hungary	72068	10 M in H_2_O	used for pH adjustment
Proteinase K	Thermo Fisher Scientific, Hungary	AM2546	20 mg/ml	5 µg/ml
Denhardt’s solution	Thermo Fisher Scientific, Hungary	750018	50×	1×
Tyramide signal amplification (TSA) fluorescence kit	Perkin Elmer, Per-Form Hungary Kft., Budapest, Hungary	NEL744001KT	Kit	Dilution factor 1:50
Anti-digoxigenin (mouse)-horse peroxidase (anti-DIG HRP) conjugate	Perkin Elmer, Per-Form Hungary Kft., Budapest, Hungary	NEF832001EA	Kit	Dilution factor 1:500
Calbindin D-28K	Swant, Switzerland	CB-38	200 µl	Dilution factor 1:1000
Calretinin	Swant, Switzerland	CG1	200 µl	Dilution factor 1:1000
Syntaxin	Santa Cruz Hungary	sc-47,437	200 µl	Dilution factor 1:100

**Table 2:** MicroRNA probes and antibodies used in the experiments

Methods	MicroRNA probe	Company	Concentration	Labelling or primary antibodies	Company		Raised in	Dilution	microRNA visualisation or secondary antibodies	Company	Raised in	Dilution
microRNA in situ detection	mir-9	Exiqon	5 or 10 nM	Anti-DIG HRP	Perkin Elmer, USA		Mouse	1:250	TSA Plus Fluorescence Cy3	Perkin Elmer, USA		1:50
	mir-23	Exiqon	10 nM	Anti-DIG HRP	Perkin Elmer, USA		Mouse	1:250	TSA Plus Fluorescence Cy3	Perkin Elmer, USA		1:50
microRNA in situ detection and single immunocytochemistry	mir-9	Exiqon	5 or 10 nM	Anti-DIG HRP	Perkin Elmer, USA		Mouse	1:500	TSA Plus Fluorescence Cy3	Perkin Elmer, USA		1:50
	mir-23	Exiqon	10 nM	Anti-DIG HRP	Perkin Elmer, USA		Mouse	1:500	TSA Plus Fluorescence Cy3	Perkin Elmer, USA		1:50
				Calbindin D-28K	Swant, Switzerland		Rabbit	1:1000	Anti-rabbit Alexa Fluor 488	Invitrogen, Hungary	goat	1:1000
				Calretinin	Swant, Switzerland		Rabbit	1:1000	anti-rabbit Alexa Fluor 488	Invitrogen, Hungary	goat	1:1000
microRNA in situ detection and double immunocytochemistry	mir-9	Exiqon	10 nM	Anti-DIG HRP	Perkin Elmer, USA		Mouse	1:500	TSA Plus Fluorescence Cy3	Perkin Elmer, USA		1:50
	mir-23	Exiqon	10 nM	Anti-DIG HRP	Perkin Elmer, USA		Mouse	1:500	TSA Plus Fluorescence Cy3	Perkin Elmer, USA		1:50
				Calbindin D-28K	Swant, Switzerland		Rabbit	1:1000	Anti-rabbit Alexa Fluor 405	Abcam, UK	donkey	1:1000
				Calretinin	Swant, Switzerland		Rabbit	1:1000	Anti-rabbit Alexa Fluor 405	Abcam, UK	donkey	1:1000
				Syntaxin	Santa Cruz Hungary		Goat	1:100	Anti-goat Alexa Fluor 488	Thermo Fisher, Hungary	Donkey	1:1000

### Equipment and preparation of reagents

RNase-free environment is essential. All equipment and working surfaces were treated with RNase Away (Thermo Fisher Scientific) or dry heat sterilization at 170 °C for at least 8 h. Caps have been soaked in DEPC-treated water overnight at 37 °C and then autoclaved. Note that solutions containing Tris cannot be treated with DEPC. When DEPC-treated solution was needed we added 1 ml of DEPC to the 1 l of solution, swirled it on a magnetic stand at room temperature for 1 h in a fume hood and autoclaved at 121 °C for 20 min. When 0.2 M phosphate buffer (PB) was needed we added 4.8 g of NaH_2_PO_4_ and 28.48 g of Na_2_HPO_4_ × 2 H_2_O to 1 l of DEPC-treated water. We made phosphate-buffered saline (PBS) from fresh PB by adding 500 ml of DEPC-treated 0.2 M PB to 500 ml of ultrapure water, dissolved 9 g of NaCl in it and autoclaved at 121 °C for 20 min. Fixative (4% PFA, pH 7.4) was prepared freshly by adding 10 g of PFA powder to heated 100 ml DEPC-treated PBS, kept at 68 °C until PFA dissolved completely, adjusted pH to 7.4 with 1 N HCl filled it up to 250 ml and finally filtered it using a 45 µm filter.

For proteinase K treatment, a specific buffer solution was prepared. Stock solutions were made of Tris–HCl (1 M, pH 7.5), NaCl (5 M) and EDTA (0.5 M) and autoclaved. To prepare proteinase K buffer, 5 ml 1 M Tris–HCl (pH 7.4), 2 ml 0.5 M EDTA and 0.2 ml 5 M NaCl were added to DEPC-treated water, the solution was filled up to 1000 ml and autoclaved at 121 °C for 20 min. To prepare proteinase K stock-solution (20 mg/ml), we dissolved 20 mg proteinase K in 1 ml 10 mM Tris–HCl (pH 7.5) and stored at − 20 °C in aliquots until use. Proteinase K working solution (5 mg/ml) was prepared from 2.5 µl proteinase K stock-solution for 10 ml proteinase K buffer. The working solution was always freshly prepared.

For acetylation, a working solution was prepared (250 µl acetic anhydride was added to 100 ml 0.1 M triethanolamine (TEA; pH 8.0) in DEPC-treated water). We added 175.3 g 3M NaCl and 88.2 g 0.3 M trisodium citrate dihydrate to 20 × saline-sodium citrate (SSC) (pH 7.0), adjusted the pH to 8.0 with 1 N HCl, filled it up to 1000 ml and treated it with DEPC.

For hybridisation (50 ml solution), we mixed 25 ml formamide, 3 ml 5 M NaCl, 1 ml 1 M Tris–HCl (pH 8.0), 0.5 ml 0.5 M EDTA, 1 ml Denhardt’s solution (50×), 25 mg yeast RNA and 18 ml DEPC-treated water and stored it at − 20 °C in aliquots. For signal detection, TSA Plus Fluorescence method was used (TSA Plus Fluorescence Kit). To enhance labelling specificity, TNB blocking was performed (TN Blocking Buffer contains 10 ml 1 M Tris–HCl (pH 7.4) and 3 ml 5 M NaCl in 100 ml ultrapure water). For preparing the blocking solution, 500 mg of blocking reagent was slowly dissolved in 100 ml heated (RNA *T*_m_ − 30 °C) TN Blocking Buffer. After blocking, washing was followed with TNT Wash Buffer (50 ml 1 M Tris–HCl (pH 7.5), 15 ml 5 M NaCl and 250 µl Tween-20 to 500 ml of ultrapure water).

## Workflow and detailed methods

**Fig. 1:**
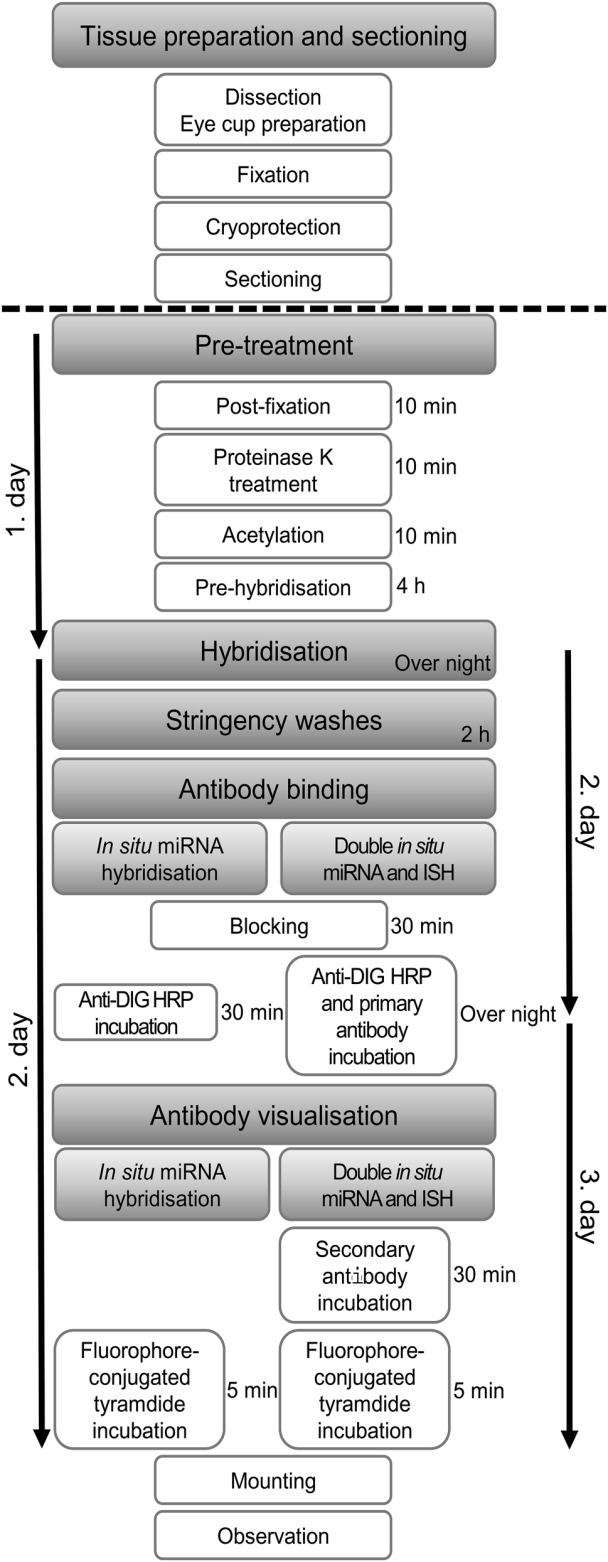
Flowchart of the protocol. The flowchart gives a brief overview of the in situ microRNA hybridisation procedure (left side), in addition to the main point of simultaneous detection of microRNA and cellular protein markers by combining microRNA in situ hybridisation with (single and double) immunocytochemistry (right side). Dashed line shows the border of the tissue preparation and labelling procedure, when a stopping point could be held and slides could be stored at − 80 °C until use

An approximate timeline and step-by-step instructions are presented in Fig. 1. All reagents and labware must be RNase-free and a particular care of preparing DEPC-treated solutions in heated sterilized glassware must be taken.

### Tissue preparation and sectioning

Animals were killed with an overdose of isoflurane anaesthetics. The dissected eyecups were immediately fixed in 4% PFA in PBS for 20 min. For cryoprotection, the tissues were placed into 15% sucrose in PBS at room temperature until the tissue sank, and then into 30% sucrose in PBS at 4 °C overnight. Next day, the cryostat was cleaned with RNase Away and sterilized with the UV-C program before usage. Then the tissues were embedded in Shandon Cryomatrix for cutting 12 µm sections and transferred to Super Frost Ultra Plus slides. Only this type of slide could be used successfully without the risk of section loss. Slides were dried for 30 min in a biological safety cabinet and were stored at − 80 °C until use.

### Pre-treatment

On the experiment day, slides were warmed to room temperature. Dried sections were fixed in 4% PFA for 10 min at room temperature and then washed three times with PBS for 5 min. To increase the permeability and the hybridisation efficiency, the proteinase K working solution was freshly prepared, the slides were treated with 5 µg/ml proteinase K for 10 min at room temperature, and finally washed three times with PBS for 5 min. During washing, the acetylation working solution could be prepared by adding 250 µl acetic anhydride with constant stirring to 100 ml 0.1 M TEA. This solution was then spread on the sections for 10 min to reduce background. This step ensured decreased binding of the negatively charged RNA probe to the acetylated negatively charged amino groups in the proteins. After the acetylation step, slides were rinsed three times with PBS for 5 min and then pre-hybridised with 700 µl hybridisation solution at room temperature for 4 h. The in situ hybridisation chamber containing a vial of 50% formamide with 1 × SSC was prepared by pre-warming to the hybridisation temperature. The hybridisation temperature was calculated from the melting temperature (*T*_m_) of the LNA-RNA probe according to manufacturer’s recommendation and set it 30 °C below the RNA *T*_m_. For instance, in our case, the RNA T_m_ of mir-9 probe was 81 °C so the hybridisation temperature was set at 51 °C, while for mir-23 probe at 53 °C.

### Hybridisation

The hybridisation mixture was prepared by adding 10 nM 5′-DIG labelled LNA-probe to hybridisation solution (the amount of the probe should be optimised). The probe was heated to 65 °C for 5 min to denature then immediately placed on ice. The pre-hybridisation buffer was replaced with the hybridisation buffer containing the appropriated amount of probe (250 µl per slide). The slides were incubated in the humidified chamber overnight at the temperatures calculated from the *T*_m_-values as indicated.

### Stringency washes

First, 5 × SSC, 0.2 × SSC solutions and 1 × SSC solution containing 50% formamide and 0.1% Tween-20 were prepared, pre-warmed above the temperature of the hybridisation solution. Slides were then washed at 5–10 °C above the hybridisation temperature once with 5 × SSC for 15 min, twice with 1 × SSC containing 50% formamide and 0.1% Tween-20 for 30 min and finally once with 0.2 × SSC for 15 min. All these steps were performed at 65 °C. Slides were then rinsed with PBS for 15 min at room temperature.

### Simultaneous antibody application for microRNA detection and immunocytochemistry

TNB Blocking solution was warmed to room temperature. Slides were incubated in TNB Blocking solutions for 30 min. The anti-DIG HRP conjugated antibody was diluted (1:250) in TNB Blocking solutions. The diluted anti-DIG HRP was administered to each slide, which were incubated for 30 min and then washed in TNT buffer three times for 5 min. When in situ hybridisation and immunostaining was performed simultaneously, the primary antibody solution was prepared in an appropriate concentration, applied together with anti-DIG HRP (1:500 dilution) and then slides were incubated overnight. Slides were rinsed in TNT buffer six times for 5 min. Species-specific secondary antibody solution (dilution 1:1000–1:10,000 depending on the make of the antibody) was prepared and slides were incubated for 2 h to visualise immunostaining, then slides were washed in TNT buffer three times for 5 min. At this point, the tyramide signal amplification (TSA) reaction was applied to reveal microRNA-s with high sensitivity. First, TSA Plus Working Solution was prepared by diluting TSA Plus Cyanine–tyramide Stock solution 1:50 in amplification buffer. Then 150 µl TSA Plus Working Solution added the slides to incubate them at room temperature for 10 min followed by washing in TNT buffer three times for 5 min. Prolong Gold containing mounting medium (with or without DAPI) was added to each slide and coverslipped. Slides were imaged the following day by a confocal microscope (40×/0.75 Ph2 UPlanFLN lens, Olympus) with the adequate laser beams.

## Results

**Fig. 2:**
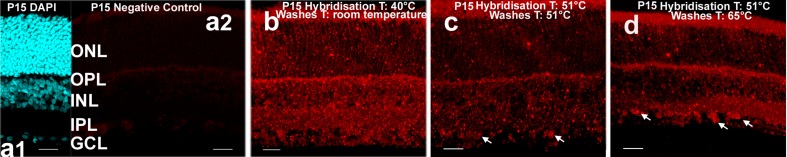
Demonstration of microRNAs (mir-9, red) in situ detection in rat retina tissues. Scale bars: 25 µm in all images (**a1, a2, b**–**d**). Relevant information regarding age and treatment conditions is shown on the images. *GCL* ganglion cell layer, *IPL* inner plexiform layer, *INL* inner nuclear layer, *OPL* outer plexiform layer, *ONL* outer nuclear layer. A false-colored (cyan) 4′,6-diamidine-2-phenylindole-dihydrochloride (DAPI) nuclear staining demonstrate the nuclear layers (**a1**), while slides incubated without microRNA probe served as negative controls (**a2**). Hybridisation was performed at 40 °C (**b**) and 51 °C (**c, d**), while stringency washes varied between room temperature (**b**), 51 °C (**c**) and 65 °C (**d**). The probe concentration was 10 nM (**b**–**d**). Note the elective labelling of the cells in the GCL (arrows in **c** and **d**)

The in situ microRNA hybridisation protocol was optimized for retinal sections from the postnatal day (PD) 7, 10, 15 and 21, which days are known to correspond with the end of cell generation, beginning of synaptogenesis, start of functional vision and completion of retinal development, respectively (Bagnoli et al. 2003; Centanin and Wittbrodt 2014). The purpose of detecting mir-9, which is known as a key regulator of the early to late developmental transition in retinal progenitors was to illustrate the appearance of a highly expressed microRNA. Incubation conditions profoundly influence labelling intensity and electivity (Fig. 2a–d), particularly the temperature of stringency washes (Fig. 2b; washing happened at the same temperature as incubation with the probe) and probe concentration (Fig. 2c, d—10 nM probe concentration; images from the 5 nM probe concentration experiments and low-temperature stringency washes are not shown because of the resulting low intensity or high background labelling). The mir-9 signal was detectable at hybridization temperatures of both 30 °C and 41 °C below its RNA *T*_m_ (81 °C) in the postnatal rat retina. Optimal labelling could be achieved using 10 nM probe concentration incubated at ~ 30 °C below RNA probe *T*_m_-value (51 °C) and stringency washes performed at 65 °C (Fig. 2d). This signal was characteristically present in the ganglion cell layer (GCL), with only a few cells showing labelling in the inner nuclear layer (INL; Fig. 2d). Further experiments have been performed using different H_2_O_2_ concentrations and proteinase K treatment schedules which also had some but not profound influence on labelling intensity (Table 3). Note that proteinase K treatment was necessary to achieve proper labelling (1 µg/ml was not enough, even with 20 min treatment time, to get satisfactory signal), however, overtreatment (10 µg/ml for 1 min) often resulted in tissue damage and section loss (Table 3). The optimum treatment was 5 µg/ml for 10 min.

**Table 3:** Outcomes of the proteinase K digestion and hydrogen peroxide (H_2_O_2_) treatment during microRNA in situ hybridisation at room temperature

Treatment	Incubation time			
	1 min	5 min	10 min	20 min
*Proteinase K* concentration				
1 µg/ml	+	+	+	−
5 µg/ml	+	++	+++	−
10 µg/ml	+	+	−	−
H_2_O_2_ concentration				
0.3%			−	
1%			−	
3%			−	

The ‘−’ sign shows negative effect of the procedure, mainly tissue damage or section loss. While ‘+’ symbols represents prosperous outcomes. The number of + signs correlates with favourable changes, such as better signal-to-noise ratio or more specific signal detection

**Fig. 3:**
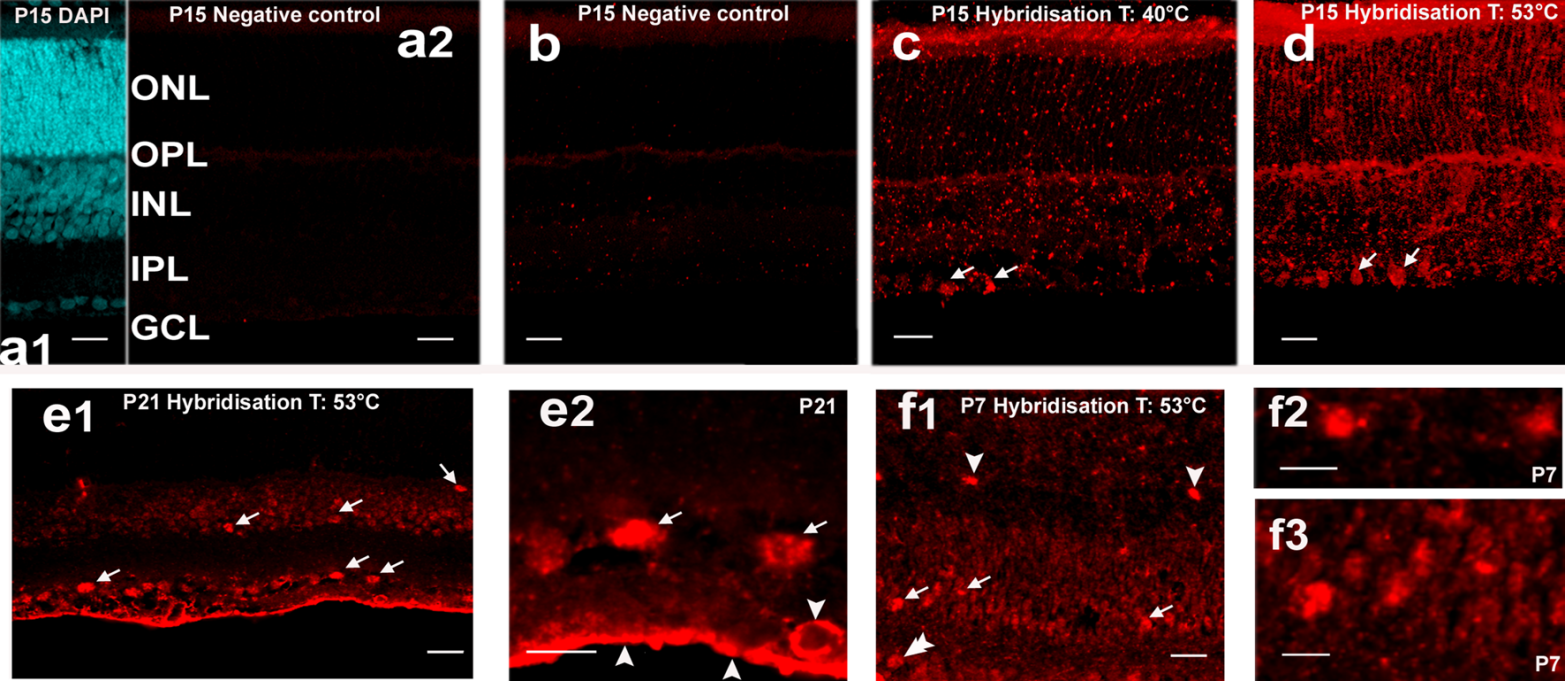
In situ detection of mir-23 in rat retina sections. *GCL* ganglion cell layer, *IPL* inner plexiform layer, *INL* inner nuclear layer, *OPL* outer plexiform layer, *ONL* outer nuclear layer, *ILM* inner limiting membrane. Relevant information regarding age and treatment conditions is shown on the images. Scale bars: 20 µm (**a1, a2, b**–**d, e1, f1**) and 10 µm (**e2, f2, f3**). For indication, the position of the nuclear layers DAPI counterstaining was applied (**a1**). The negative controls (**a2, b**) were obtained with sections incubated in the absence of both the mir-23 probe and the anti-DIG-horseradish peroxidase (HRP)-labelled antibody (**a2**); to detect the specificity of tyramide signal amplification sections were incubated without probe but with anti-DIG HRP antibody and tyramide signal amplification system (**b**). Representative images of mir-23 hybridised at 40 °C (**c**) and 53 °C (**d)**; arrows indicate labelled cells. Note the more prominent staining in **d**. Strong mir-23 labelling can be seen in the cells of INL and GCL at P21 at 53 °C hybridisation temperature (**e1**—arrows). Presumed ganglion cells are especially strongly stained (**e2**—arrows). Non-specific staining of the inner limiting membrane and a capillary is indicated with arrowheads. As early as P7, the mir-23 signal can already be detected in several retinal cell types (**f1**) including horizontal cells (arrowheads), amacrine cells (arrows) and a presumed ganglion cell (double arrowhead). Enlarged image of horizontal (**f2**) and amacrine (**f3)** cells, respectively

The microRNA mir-23 is a potential regulator in the development of the GABAergic system that is particularly elaborate in the retina (Yang 2004). In our mir-23 experiments (Fig. 3) expression was observed best at 53 °C hybridisation temperature. Signal could not be observed in sections incubated without microRNA probe and anti-DIG-HRP or slides incubated with anti-DIG HRP only (Fig. 3a2, b, respectively), both tests served as negative controls. Just like in the case of mir-9, mir-23 label was detected most prominently in the GCL (Fig. 3c, d). Hybridisation temperature had a profound effect on labelling intensity; at 40 °C staining was less intense (Fig. 3c) while 53 °C (calculated from *T*_m_) yielded the best results (Fig. 3d). Besides cells in the GCL (Fig. 3e1, e2), staining was observed for perhaps all horizontal (Fig. 3f1, f2) and some amacrine cells (Fig. 3 e1, f). The inner limiting membrane and some capillaries occasionally showed non-specific staining (Fig. 3e2). To optimise the probe concentration, values ranging from 1 to 20 (1, 2, 5, 10, 20) nM were tested and specific signal was obtained with various intensities and backgrounds (images not shown). The optimal probe concentration was 10 nM for this microRNA, similarly to mir-9.

**Fig. 4:**
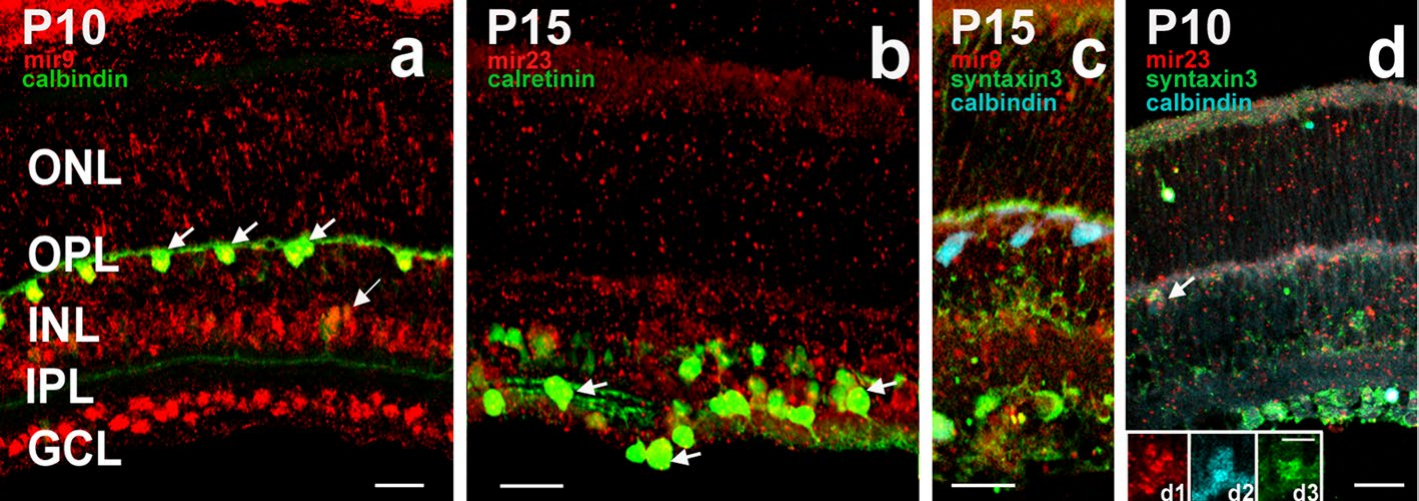
Co-detection of microRNAs (mir-9, mir-23), neuron-specific marker protein (calbindin D28k or calretinin) and synaptic marker protein (syntaxin-3), respectively. *GCL* ganglion cell layer, *IPL* inner plexiform layer, *INL* inner nuclear layer, *OPL* outer plexiform layer, *ONL* outer nuclear layer. Relevant information regarding age and treatment conditions is shown on the images. Scale bars: 25 µm (**a**–**d**) and 5 µm (**d1**–**d3**). In situ hybridization detection of mir-9 (red—Cy3) showed specific co-labelling in all horizontal cells (green—Alexa Fluor 488; calbindin D28k, marked with arrows), while only a few amacrine cells (one indicated with arrow) were double labelled for mir-9 and calbindin. Cells in the GCL were labelled invariably for mir-9 only (**a**). Dual ISH-IHC of mir-23 (red—Cy3) and calretinin (green—Alexa Fluor 488) was detected mainly in the amacrine cells and presumed ganglion cell bodies in the GCL (**b**). A few double-labelled cells are marked with arrows. A representative image showing the in situ hybridisation signal (red: mir9—Cy3) combined with double immunocytochemical labelling (blue: calbindin—Alexa Fluor405 and green: syntaxin-3 - Alexa Fluor 488) (**c**). Demonstration of triple labelling in P10 retinae. The in situ hybridisation signal (red: mir23—Cy3) combined with double immunocytochemical labelling (blue: calbindin—Alexa Fluor 405 and green: syntaxin-3—Alexa Fluor 488). A triple-labelled horizontal cells is marked with arrow (**d**). Insert images demonstrate the overlap of ISH-dual IHC staining in a horizontal cell. The red signal (Cy3) is for mir-23 (**d1**), blue signal (Alexa Fluor 405) is for calbindin (**d2**) and green signal (AlexaFluor 488) is for syntaxin-3 (**d3**), respectively

For further improvement of the methodology, microRNA in situ hybridisation was combined with immunocytochemistry (Fig. 4). The immunocytochemical procedure was integrated into the microRNA in situ hybridisation protocol, thus making possible to perform the multiple labelling protocol in 3 days. After the probe hybridisation and stringency wash steps, a common blocking step was performed for probe detection and immunocytochemistry, then anti-DIG HRP was applied simultaneously with the primary antibody of the immunocytochemical procedure. Combination of the two methodologies after our mild proteinase K treatment protocol became possible, and was useful for identifying cells expressing a particular microRNA and a calcium-binding protein (Fig. 4a, b—calbindin and calretinin, respectively). Stronger proteinase K treatment (longer incubation times, higher concentrations and/or higher temperature) resulted in the loss of antigenicity of the targeted protein markers thus making the simultaneous detection impossible (images not shown). To take this methodological improvement further we lastly decided to apply our protocol for simultaneous detection of mir-9, calbindin (a horizontal cell-specific marker in the retina) and syntaxin-3, a synaptic protein marker (Fig. 4c). We were successful in detecting these three markers in the horizontal cells (Fig. 4d). The distribution of the reaction product within the same cell is slightly different (Fig. 4d1–d3), clearly indicating that the labels are specific and we found no evidence of any false positivity in our triple labelling protocol.

## Discussion

In the present study, we described a detailed method for sensitive histological detection of microRNAs with simultaneous single or double immunocytochemical labelling. Several papers have reported microRNA in situ hybridisation detection (de Planell-Saguer et al. 2010; Kasai et al. 2016), either in frozen tissues (Obernosterer et al. 2007; Silahtaroglu et al. 2007) and/or in retina sections (Thompson et al. 2007; Singh et al. 2014). Double labelling protocols mostly focused on microRNA–mRNA co-detection (Kasai et al. 2016) and only very few papers have intended to combine microRNA in situ hybridisation with immunolabelling (Singh et al. 2014). The main advantages of our method can be summarised as follows: (1) our proteinase K treatment protocol makes possible a better probe penetration then in the previous papers but at the same time it does not destroy protein antigens allowing for the simultaneous immunocytochemical detection of cellular proteins; (2) hybridisation and stringency washes at different temperatures, calculated from the *T*_m_ of the actual probe, result in stronger and more elective labelling, and (3) a better cellular resolution of the microRNA labelled cells. To our knowledge, this study is the first that aimed at to localise a microRNA expression with two protein markers at the same time in frozen retinal sections. Thus, this technique could be suitable for simultaneous detection of a particular microRNA and a potentially associated target protein in a cell that is also identified by its characteristic cellular marker protein. In view of these results through this protocol, we expect to get a deeper insight into retinal physiological processes such as cellular proliferation, differentiation or apoptosis.

For sensitive and highly specific microRNA in situ hybridisation, several challenges had to be overcome (Urbanek et al. 2015). The first crucial steps are tissue preparation and pre-treatment, which influenced the detectability remarkably in the case of these small RNA molecules. For frozen retinal sections, generally a rapid (30 min–2 h) 4% paraformaldehyde fixation has been used. In contrast to this, various descriptions can be found for pre-treatments that differ significantly in the order of the steps, the treatment/incubation time or applied concentration of chemicals. These steps serve to enhance probe penetration and target sequence matching. To improve permeabilisation or to remove protein that surrounds the target sequence some protocols prescribe proteinase K treatment (Obernosterer et al. 2007; Silahtaroglu et al. 2007; Thompson et al. 2007), whereas others do not (Song et al. 2010; de Planell-Saguer et al. 2010; Nielsen et al. 2014). Our experiments confirm that proteinase K treatment is essential for elective microRNA detection, but it demands special caution since overtreatment may cause tissue damage and section loss. Therefore, optimal concentration and incubation time of proteinase K treatment should be determined for each tissue type. Another key step is acetylation to decrease background, however, most protocols do not vary significantly in this respect including our currently suggested method. On the other hand, the tyramide signal amplification method that uses horseradish peroxidase to visualize microRNA labelling is a sensitive step. Using peroxidase enzyme makes it essential to eliminate the endogenous tissue peroxidase activity which could result in a very high background. For this reason, H_2_O_2_ treatment is generally used as a pre-treatment step. We found that this procedure could cause serious tissue degradation in certain conditions, so we minimised this step in our protocol. The most critical steps, however, are the hybridization temperature and the stringency washes. They both have crucial effects on the intensity and specificity of in situ hybridisation signals. Karali and co-workers in their comprehensive study reported a detailed description about the applied hybridisation temperatures for the different probes (Karali et al. 2010). As mentioned in their work 40 °C was applied for mir-9 and mir-23 hybridisation, but our study demonstrates that this condition could give a high background staining. In contrast, the manufacturer recommends using the miRCURY LNA probes at 30 °C below the microRNA *T*_m_, so we calculated the hybridisation temperature from the probe sequence. Our examination confirmed that the hybridisation at 51 °C and 53 °C was most effective for specific detection of mir-9 and mir-23, respectively. However, the signal-to-noise ratio even then could be non-satisfactory, so optimisation of another important parameter, the temperature of stringency washes was necessary to avoid false-positive signals. When the wash steps following the hybridisation procedure were carried out less than or equal to hybridisation temperature it caused very strong false-positive and/or background signals. In contrast, hybridisation at a higher temperature could even destroy the specific probe–target hybridisation and even eliminate the signal. The best signal-to-noise ratio could be achieved when hybridisation was performed at 30 °C below the microRNA *T*_m_ (as probe manufacturer’s offers) followed by stringency washes at 5–10 °C higher temperature.

In conclusion, this paper presents a refined method for visualizing microRNA expression. Successful combination of this method with immunocytochemical detection of proteins provides possibilities to get a deeper insight into the gene regulation networks during retinal histogenesis. Since in situ hybridisation serves information on spatial distribution of the microRNAs, this technique cannot be used for quantitative determination. Our ongoing investigations focus on the expression profile of retinal microRNAs in development and quantify the most important players by next-generation sequencing and quantitative PCR measurements.

## Abbreviations

Anti-DIG HRP: Anti-digoxigenin (mouse)-horseradish peroxidase
DEPC: Diethylpyrocarbonate
Na_2_HPO_4_ × 2 H_2_O: Di-sodium hydrogen phosphate dehydrate
EDTA: Ethylenediaminetetraacetic acid
HCl: Hydrochloric acid
ISH: In situ hybridisation
PFA: Paraformaldehyde
PB: Phosphate buffer
PBS: Phosphate-buffered saline
SSC: Saline-sodium citrate
NaH_2_PO_4_: Sodium-dihydrogen phosphate anhydrous
TEA: Triethanolamine
TSA: Tyramide signal amplification
